# A Method for Measuring Parameters of Defective Ellipse Based on Vision

**DOI:** 10.3390/s23146433

**Published:** 2023-07-15

**Authors:** He Zhang, Li Wang, Wenya Liu, Jiwen Cui, Jiubin Tan

**Affiliations:** 1 Center of Ultra-Precision Optoelectronic Instrument, Harbin Institute of Technology, Harbin 150080, China; 15546366118@163.com (H.Z.); wang_li2022@163.com (L.W.); 17863964533@163.com (W.L.); jbtan@hit.edu.cn (J.T.); 2 Key Lab of Ultra-Precision Intelligent Instrumentation, Harbin Institute of Technology, Ministry of Industry and Information Technology, Harbin 150080, China

**Keywords:** visual measurement, defect ellipse repair, outlier removal, least squares fitting

## Abstract

Ellipse detection has a very wide range of applications in the field of object detection, especially in the geometric size detection of inclined microporous parts. However, due to the processing methods applied to the parts, there are certain defects in the features. The existing ellipse detection methods do not meet the needs of rapid detection due to the problems of false detection and time consumption. This article proposes a method of quickly obtaining defective ellipse parameters based on vision. It mainly uses the approximation principle of circles to repair defective circles, then combines this with morphological processing to obtain effective edge points, and finally uses the least squares method to obtain elliptical parameters. By simulating the computer-generated images, the results demonstrate that the center fitting error of the simulated defect ellipses with major and minor axes of 600 and 400 pixels is less than 1 pixel, the major and minor axis fitting error is less than 3 pixels, and the tilt angle fitting error is less than 0.1°. Further, experimental verification was conducted on the engine injection hole. The measurement results show that the surface size deviation was less than 0.01 mm and the angle error was less than 0.15°, which means the parameters of defective ellipses can obtained quickly and effectively. It is thus suitable for engineering applications, and can provide visual guidance for the precise measurement of fiber probes.

## 1. Introduction

With the development of the precision manufacturing industry and the increasing demand for technical indicators, micropore array structures combining the macro and micro scales have emerged in various high-performance devices, such as micropore arrays on aircraft engine injection disks with over 4700 micropores. The shape and size of the pores have a significant impact on the performance of the parts [1]. In the process of processing and usage, there may also be defects on the surfaces of micropores. The quality of microporous structure processing will determine the quality of the components, so the rapid detection of parameters is of great significance. Image methods, due to their ability to quickly obtain data, have great potential for use in visual measurement or multi-sensor fusion parameter measurement.

For a spatial aperture, when the imaging plane is not parallel to the plane where the aperture is located, the projection of the formed image on the camera imaging plane takes the form of an ellipse. The shape and size of the circular hole can be transformed from an ellipse, which means accurate acquisition of elliptical parameters is crucial. There are three general ellipse fitting algorithms, namely, the method of least squares, the method based on Hough transform, and the method based on arc segment extraction.

Ellipse fitting based on the least squares method is an optimal estimation technique that uses the maximum likelihood method to minimize the sum of squares of measurement errors when the random error is normal distribution. However, it is greatly affected by outliers, so the focus of improvements is to remove outliers. Li [2] used the double segmentation method to segment two overlapping ellipses to obtain two sets of effective ellipse edge points. Maini [3] proposed an improved ellipse direct least squares fitting method, which utilized normalization techniques and introduced disturbance and resampling strategies to make it superior in terms of robustness, but it increased the computational burden. Liang [4] constructed a new non-convex nonlinear optimization formula to improve the ability to suppress outliers, but it also has the problem of low computational efficiency. Azarakhsh [5] proposed a real-time ellipse detection method, which first extracts all contours from the input image using the ad hoc method, and then performs ellipse fitting on the contours using the least squares method. However, this method achieves lower accuracy in detecting elliptical targets with short distance.

The second ellipse-fitting method is the method based on Hough transform. Its basic idea is consistent with Hough line detection, which first maps the data to a specific parameter space and then votes to obtain the ellipse parameters. However, due to the fact that the ellipse has five free parameters, the time and memory costs are unacceptable. Some scholars have also proposed improved Hough ellipse detection algorithms. The usual approach is to use the geometric properties of ellipses and edge direction information for parameter decomposition [6,7,8], but this still requires a lot of time and memory. Xu [9] proposed a random Hough transform method to reduce the memory requirement. Some scholars have applied this in ellipse detection [10,11], which requires five points to determine the ellipse parameters. Chen [12] Yanxin proposed a random Hough transform ellipse detection method that only needs three points to determine the ellipse parameters, but it is still time-consuming. Subsequently, Kwon [13] proposed a method for the rapid detection of ellipses using the geometric characteristics of three points on an ellipse, using the normal and differential equations of the ellipse. This method achieved good results in both detection reliability and computational speed. Yasuaki [14] decomposed the parameter space in the Hough transform into each parameter, and performed serial calculations on each parameter, which were implemented on a GPU system to reduce computational time and space.

The third ellipse fitting method is based on arc segment extraction, which has been proposed in recent years, and the basic idea is to replace discrete edge points with a set of continuous edge points named arc segment that form an ellipse as ellipse detection data. Prasad and Leung used elliptical arcs to fit ellipses, and through fitting calculations, the center of the ellipse can be obtained [15]. Afterwards, Wang adopted a top-down strategy by merging edge points into ellipses and using an integral chain to accelerate the fitting process [16]. Lu proposed a method based on arc-supported line segments, which uses the Line Segment Detector (LSD) algorithm for arc segment extraction and has high detection accuracy [17,18]. Meng used the arc segment adjacency matrix to obtain a method for combining all arc segments. This construction method will consume a lot of computer memory [19].

Due to the existence of defects, the current ellipse detection methods have significantly worse fitting performance and poor robustness. Moreover, in order to ensure the accuracy of visual measurement, the size of the image obtained by vision is too large. This greatly increases the time and cost. For the combination of array structure vision and probe measurement, the imaging time of a single hole feature in the field of view is limited [20], so there is a requirement related to the fitting time of elliptical features. Moreover, the existing processing methods do not fully consider the influence of elliptical defects and interference points, resulting in a decrease in the accuracy of ellipse fitting. As such, this article proposes an edge repair algorithm based on the approximation principle of circles to repair defect ellipses, thereby proposing outliers, and the image repaired by the least squares method is used for ellipse fitting, which can obtain ellipse parameters quickly and accurately.

## 2. Measurement Principles and Analysis

### 2.1. Camera Imaging Model

**Figure 1:**
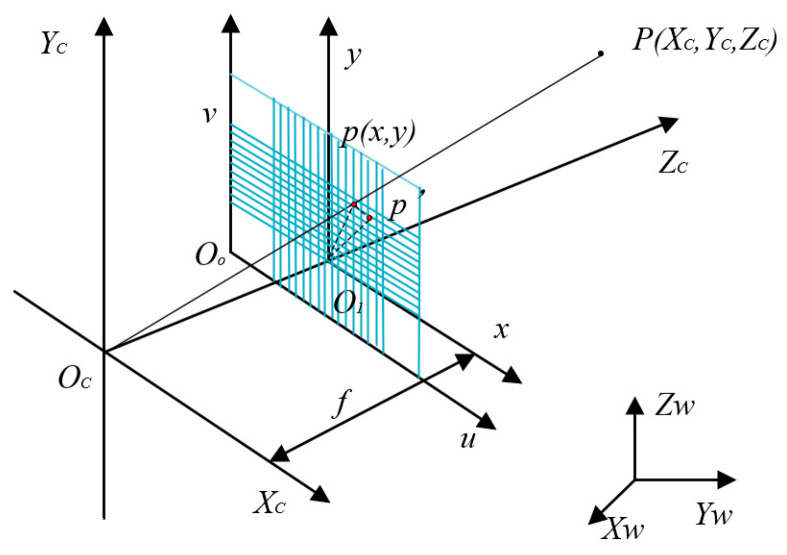
Camera imaging model.

For the aperture to be measured, the camera is first used to obtain an image of the micropores. The projected image is an ellipse on the camera’s imaging plane. To obtain the geometric information of the micropores, a perspective projection model of the geometric correspondence between the image points and the spatial object points needs to be established. The projection position p of any point P on the micropore in the image is the intersection of the line connecting the optical center O and point P with the image plane, as shown in Figure 1.

O_w_-X_w_Y_w_Z_w_ is the world coordinate system. O_c_-X_c_Y_c_Z_c_ is the camera coordinate system. O_1-xy_ is the imaging plane coordinate system. O_o-uv_ is the pixel coordinate system. The coordinates of point P in the world coordinate system are (X_wp_, Y_wp_, Z_wp_). The coordinates in the camera coordinate system are (X_cp_, Y_cp_, Z_cp_). The coordinates of point p in the imaging plane coordinate system are (x_p_, y_p_). In the pixel coordinate system, the coordinates are (u_p_, v_p_). The formula of conversion from the world coordinate system point P_w_ (X_w_, Y_w_, Z_w_) to the camera coordinate system imaging point P_c_ (X_c_, Y_c_, Z_c_) is expressed as the following formula, where R is a rotated orthogonal matrix and T is a translation matrix:

(1) $$\left[\begin{array}{c} X_{c}\\ Y_{c}\\ Z_{c}\\ 1 \end{array}\right]=\left[\begin{array}{cc} R & T\\ 0^{T} & 1 \end{array}\right]\left[\begin{array}{c} X_{w}\\ Y_{w}\\ Z_{w}\\ 1 \end{array}\right]$$

In the camera coordinate system, the optical center O_c_ of the camera is the origin, the Z_c_ axis is the optical axis, the X_c_ axis is parallel to the imaging coordinate system’s x-axis, and the Y_c_ axis is parallel to the imaging coordinate system’s y-axis. The vertical distance from the optical center O_c_ to the imaging plane O_1−xy_ is the effective focal length f. According to the projection principle, the conversion model between the camera coordinate system and the imaging coordinate system can be expressed as follows:

(2) $$\left\{\begin{array}{c} x=f\frac{X_{c}}{Z_{c}}\\ y=f\frac{Y_{c}}{Z_{c}} \end{array}\right.$$

The matrix form can be expressed as follows:

(3) $$Z_{c}\left[\begin{array}{c} x\\ y\\ 1 \end{array}\right]=\left[\begin{array}{cccc} f & 0 & 0 & 0\\ 0 & f & 0 & 0\\ 0 & 0 & f & 0 \end{array}\right]\left[\begin{array}{c} X_{c}\\ Y_{c}\\ Z_{c}\\ 1 \end{array}\right]$$

The coordinates of the origin O_1_ of the imaging plane coordinate system in the pixel coordinate system are (u_0_, v_0_), where dx and dy, respectively, represent the actual size of each pixel in the x and y directions. The point coordinates in the pixel coordinate system can be represented as follows:

(4) $$\left\{\begin{array}{c} u=\frac{x}{\mathrm{dx}}{+u}_{0}\\ v=\frac{y}{\mathrm{dy}}{+v}_{0} \end{array}\right.$$

The matrix form of homogeneous coordinates is:

(5) $$\left[\begin{array}{c} u\\ v\\ 1 \end{array}\right]=\left[\begin{array}{ccc} 1/d_{x} & 0 & u_{0}\\ 0 & 1/\mathrm{dy} & v_{0}\\ 0 & 0 & 1 \end{array}\right]\left[\begin{array}{c} x\\ y\\ 1 \end{array}\right]$$

From (1) to (5), it can be concluded that the relationship between the camera coordinate system and the pixel coordinate system is:

(6) $$Z_{c}\left[\begin{array}{c} u\\ v\\ 1 \end{array}\right]=\left[\begin{array}{ccc} 1/d_{x} & 0 & u_{0}\\ 0 & 1/\mathrm{dy} & v_{0}\\ 0 & 0 & 1 \end{array}\right]\left[\begin{array}{cccc} f & 0 & 0 & 0\\ 0 & f & 0 & 0\\ 0 & 0 & f & 0 \end{array}\right]\left[\begin{array}{c} X_{c}\\ Y_{c}\\ Z_{c}\\ 1 \end{array}\right]$$

Next, we can obtain the correspondence between the world coordinate system point Pw and the pixel coordinate system point p:

(7) $$Z_{c}\left[\begin{array}{c} u\\ v\\ 1 \end{array}\right]=\left[\begin{array}{ccc} f_{x} & 0 & u_{0}\\ 0 & f_{y} & v_{0}\\ 0 & 0 & 1 \end{array}\right]\left[\begin{array}{cc} R & T\\ 0^{T} & 1 \end{array}\right]\left[\begin{array}{c} X_{w}\\ Y_{w}\\ Z_{w}\\ 1 \end{array}\right]$$

We then normalize the focal lengths in the x and y directions in the equation, where f_x_ and f_y_ are equivalent focal lengths (f_x_ = f/d_x_, f_y_ = f/d_y_).

### 2.2. Ellipse Fitting Based on Least Squares Method

Ellipse fitting based on the least squares method is an optimal estimation technique derived from the maximum similarity method when the random error is normal distribution [21], which reduces the sum of distances between all points to be fitted and the fitting curve to its minimum. The general form of an ellipse is:

(8) $${\mathrm{Ax}}^{2}{+\mathrm{Bxy}+\mathrm{Cy}}^{2}+\mathrm{Dx}+\mathrm{Ey}+1=0$$

So the goal of the least squares method is to minimize the objective function f(A, B, C, D, E), which is defined as follows:

(9) $$f(A,B,C,D,E)=\sum_{i=1}^{n}({\mathrm{Ax}}_{i}^{2}{+\mathrm{Bx}}_{i}y_{i}{+\mathrm{Cy}}_{i}^{2}{+\mathrm{Dx}}_{i}{+\mathrm{Ey}}_{i}+1{)}^{2}$$

Then, the linear function is constructed through the extreme value theorem, and the ellipse parameters are solved by combining the constraint conditions. From the geometric properties of the ellipse, the parameters are:

(10) $$\left\{\begin{array}{l} x_{e}=\frac{\mathrm{BE}-2\mathrm{CD}}{4\mathrm{AC}-B^{2}}\\ y_{e}=\frac{\mathrm{BD}-2\mathrm{AE}}{4\mathrm{AC}-B^{2}}\\ a=\sqrt{\frac{{2(\mathrm{Ax}}_{e}{}^{2}{+\mathrm{Cy}}_{e}{}^{2}{+\mathrm{Bx}}_{e}y_{e}-1)}{A+C+\sqrt{{(A-C)}^{2}{+B}^{2}}}}\\ b=\sqrt{\frac{{2(\mathrm{Ax}}_{e}{}^{2}{+\mathrm{Cy}}_{e}{}^{2}{+\mathrm{Bx}}_{e}y_{e}-1)}{A+C-\sqrt{{(A-C)}^{2}{+B}^{2}}}}\\ \varphi =\frac{1}{2}\arctan\frac{B}{A-C} \end{array}\right.$$

Among them, (x_e_, y_e_) represents the center of the ellipse. (a, b) represent the major and minor axes of the ellipse, and φ is the inclination angle.

According to the principle of the least square method, too many outliers can lead to the random error not conforming to normal distribution, which will greatly affect the fitting effect.

### 2.3. A Defect Edge Repair Model and Evaluation Based on Approximate Circles

**Figure 2:**
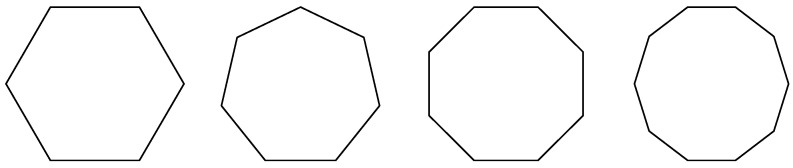
Approximation of circles.

In the actual production and manufacturing of precision components, machining errors such as burrs may occur in the circular features to be measured due to processing methods, or significant errors may occur in the binary image results due to surface indentation. This can affect measurement accuracy during data fitting. The repair method for defective circles is mainly based on the principle that circles can be approximated as regular n-sided shapes. The schematic diagram is shown in Figure 2.

**Figure 3:**
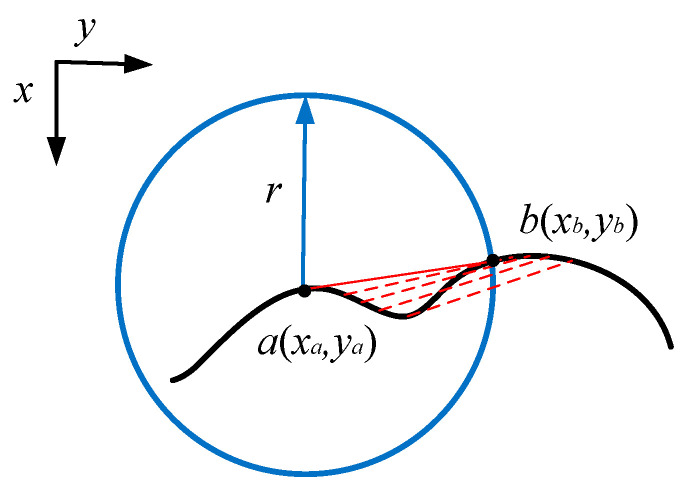
Repair of circles.

Based on this issue, this article proposes a circular edge repair method based on the principle of circle approximation. It is based on the edge sequences of a circle obtained through edge detection after binarization, and connects the edge sequence points in a straight line to remove the protruding parts. Finally, the region with the largest connected domain is taken as the repaired feature area. The schematic diagram of edge repair is shown in Figure 3.

Firstly, the method obtains an ordered edge sequence of features from the feature map that need to be repaired, and then traverses the edge sequence so that the traversed edge point is a (x_a_, y_a_), and its related point b (x_b_, y_b_) is the edge point with a sequence number after it, and the following conditions are met:

(11) $$\sqrt{{{(x}_{a}-x_{b})}^{2}{+(y}_{a}-y_{b}{)}^{2}}-r\leq 1$$

R is the repair radius. The edge repair algorithm is:

(12) $${c(x}_{c}{,y}_{c})=0,\;\frac{\left|{(y}_{a}-y_{b}{)x}_{c}{+(x}_{b}-x_{a}{)y}_{c}{+x}_{a}y_{b}-x_{b}y_{a}\right|}{\sqrt{{{(y}_{a}-y_{b})}^{2}{+(x}_{b}-x_{a}{)}^{2}}}\leq 1$$

(13) $$\left\{\begin{array}{l} {\min(x}_{a}{,x}_{b}{)\;<\;x}_{c}{<\;\max(x}_{a}{,x}_{b})\\ {\min(y}_{a}{,y}_{b}{)\;<\;y}_{c}{<\;\max(y}_{a}{,y}_{b}) \end{array}\right.$$

**Figure 4:**
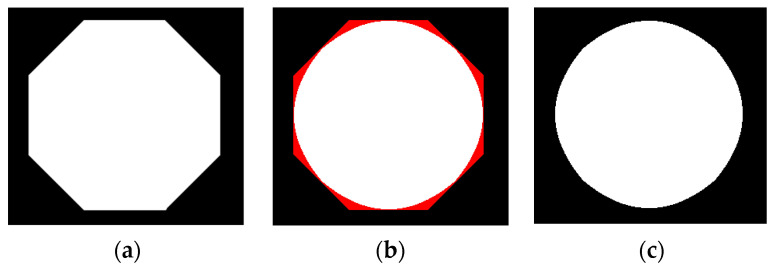
Repair of a regular polygon. (**a**) Original image. (**b**) Repair result graph. (**c**) Cutting result graph.

C(x_c_, y_c_) is the repair point, with its physical meaning being points a and b forming a single point on the line segment. We used the above method to process a regular hexagon, and included the Bresenham algorithm of computer graphics in the method’s implementation when cutting. The results are shown in Figure 4.

From Figure 4, it can be seen that this method is based on the approximation principle of circles. The cutting part is shown in red in Figure 4b, and the repair result is shown in Figure 4c. It can be seen that this method can effectively cut the features of the approximate circle into circles, that is, repair the shape of the approximate circle in circles. The same applies when detecting an ellipse. After multiple repairs, a smooth ellipse can be obtained, which can be used as an approximate circular target. Therefore, the limit on the number of repairs can be evaluated using circularity. The definition of circularity a is [22]:

(14) $$a=\frac{4\pi S}{C^{2}}$$

Among them, S is the area of the target and C is the circumference of the target. When the target shape is closer to a circle, its value is closer to 1. Therefore, whether the difference in roundness between the two repairs is less than the given threshold T is used to determine whether the repair is completed.

After obtaining the target that approximates a circle, a morphological dilation algorithm is used to process the repaired image. During each dilation process, the intersection of the repaired and original images’ edge points is recorded, and the part where the number of intersecting edge points increases due to each dilation is recorded. When the proportion of intersecting edge points exceeds the threshold, the dilation operation is stopped, and the edge point showing the largest increase in the number of intersecting points is taken as the effective edge point. By performing least squares ellipse fitting as described in the second section, the ellipse parameters can be obtained.

## 3. Experiment and Verification

### 3.1. Simulation Experiment

**Figure 5:**
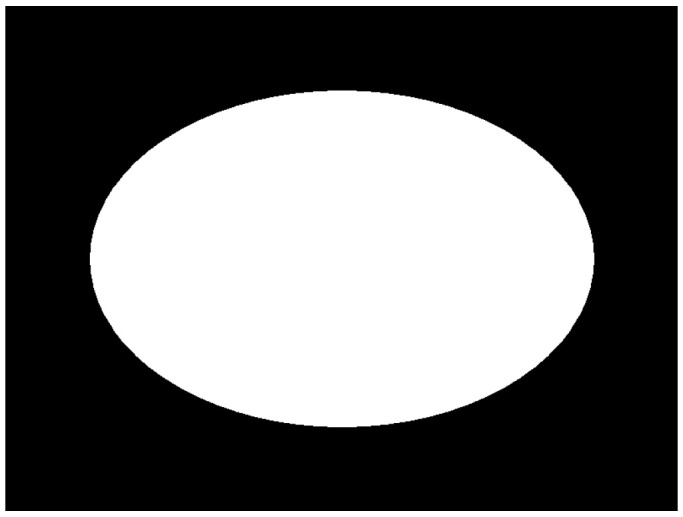
Standard ellipse.

To simulate and validate the method proposed in this article, a computer is first used to generate an ellipse with known parameters, as shown in Figure 5. The center position of the ellipse is (300, 400), the major and minor axes are (600, 400), and the tilt angle is 0 degrees.

**Figure 6:**
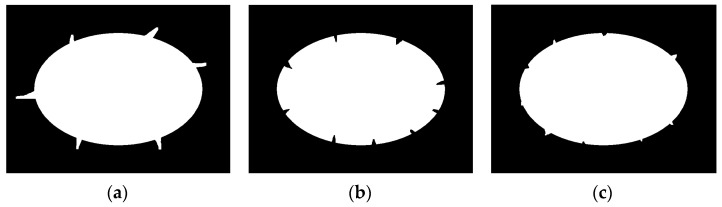
Defect ellipse. (**a**) Protruding defects; (**b**) concave defects; (**c**) mixed defects.

We then add three types of defects to it. As shown in Figure 6, there are protruding defects, concave defects, and mixed defects.

**Figure 7:**
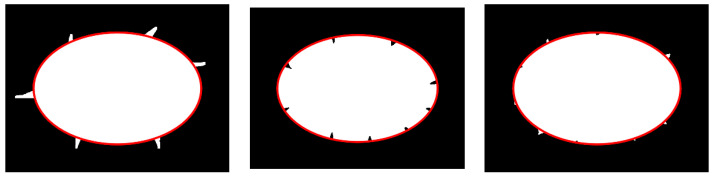
Defect ellipse fitting results.

The fitting results obtained by using the edge repair fitting method mentioned in this article are shown in Figure 7.

Their respective fitting parameter results are shown in Table 1.

From the simulation results shown in Figure 7 and Table 1, it can be seen that the method proposed in this article can effectively eliminate convex outliers and concave outliers, and obtain accurate ellipse parameters. The center fitting error of the generated defect ellipse with a major and minor axis of 600 and 400 pixels is less than 1 pixel, the major and minor axis fitting error is less than 3 pixels, and the tilt angle fitting error is less than 0.1°.

### 3.2. Experimentation

**Figure 8:**
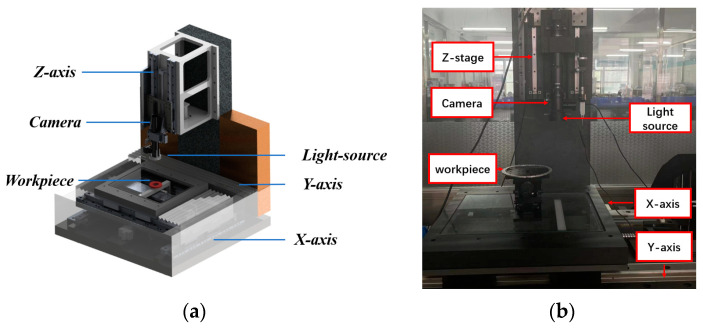
Measurement system. (**a**) A 3D diagram of the measurement system; (**b**) a physical diagram of the measurement system.

**Figure 9:**
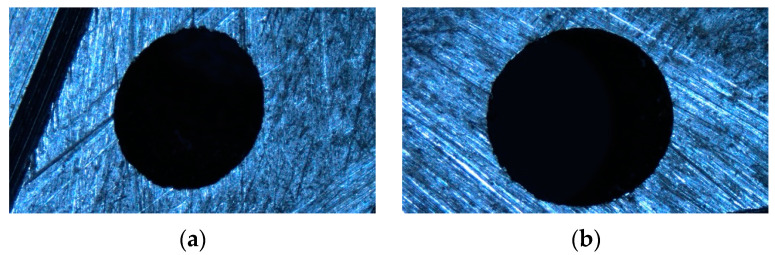
Tested oblique hole image: (**a**) small hole; (**b**) large hole.

In order to verify the effectiveness of the algorithm when used in practical applications, the measurement system shown in Figure 8a is used to obtain part images. The base and *Z*-axis column of the measurement system are made of marble to ensure the stability of the system. The workbench can move along the x, y, and z axes, and there is a camera installed on the z axis. A PointGray model FL3-U332S2C-CS CCD camera (Point Grey Research, Richmond, BC, Canada) is used, and the NAVITAR 12× zoom combination lens is used. After calibration, the average reprojection error is about 0.05 pixels. The measured parts are two sub-millimeter inclined micropores. The images of large and small holes with defect features are shown in Figure 9, where elliptical defects can be clearly seen.

**Figure 10:**
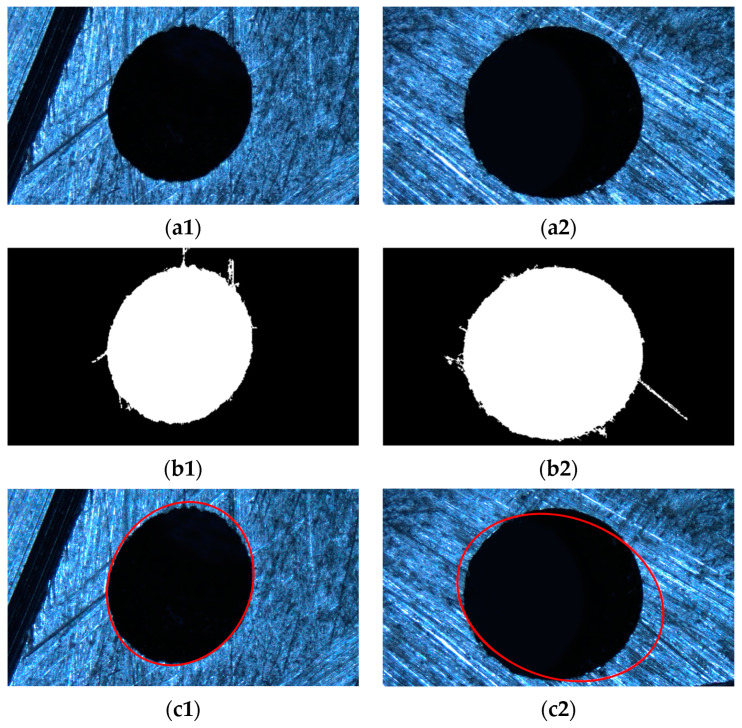
Defect micropore characteristics. (**a1**) Aerospace engine injection disk micropores (small). (**a2**) Aerospace engine injection disk micropores (large). (**b1**) Binary graph (small). (**b2**) Binary graph (large). (**c1**) Direct least squares fitting (small). (**c2**) Direct least squares fitting (large).

Firstly, an image segmentation method based on image gradient information is used to obtain the elliptical feature positions for the part image. Then, based on the microhole’s features, they are binarized. The results are shown in Figure 10b.

**Figure 11:**
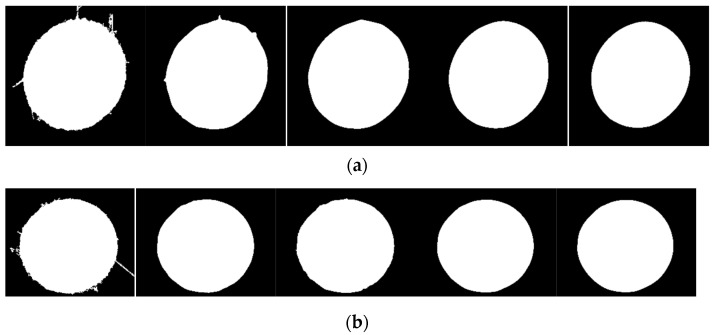
Repair of defective circles. (**a**) Small hole; (**b**) large hole.

For defect images, certain methods must be used to remove the edge of the defect, otherwise fitting it will result in incorrect fitting results, as shown in Figure 10c. Therefore, this article proposes a defect circle repair method, as shown in Section 2, which repairs the binary image shown in Figure 10b multiple times, as shown in Figure 11. For the repair of defect images, the roundness threshold for the repair cutoff is set as 1.24, based on the experimental results. Finally, both the small and large holes were repaired up to the fourth stop.

Next, we performed a dilation operation on the binarized image, and a schematic diagram of the dilation operation is shown in Figure 11.

**Figure 12:**
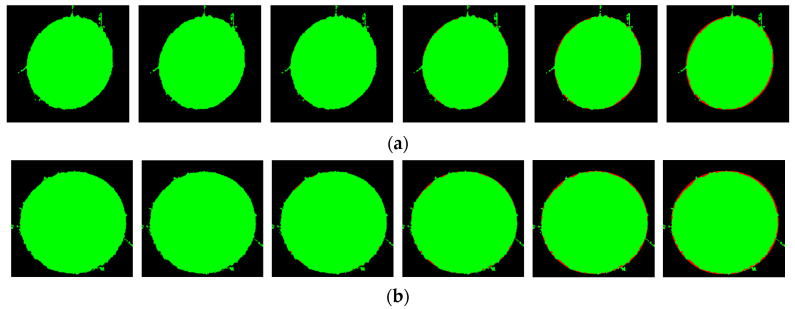
Schematic diagram of the difference between the repaired image and the original image. (**a**) Small hole; (**b**) large hole.

**Figure 13:**
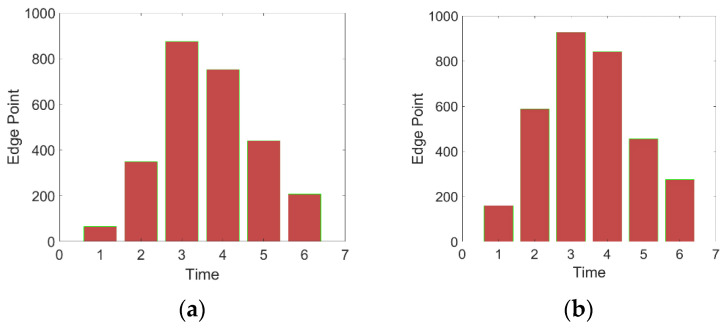
New intersection edge points added after expansion. (**a**) Small hole; (**b**) large hole.

Figure 12 represents the difference between the corresponding repaired image and the original image to indicate their intersection. The green part represents the difference between the original image and the repaired image, and the red part represents the difference between the repaired image and the original image. The increase in the number of edge points at each intersection is shown in Figure 13.

**Figure 14:**
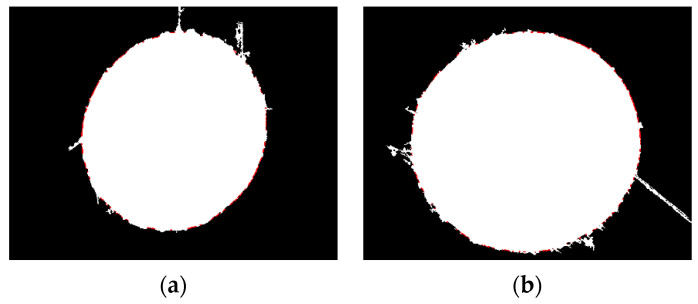
Effective edge point of defect ellipse. (**a**) Small hole; (**b**) large hole.

From the bar chart, for small holes, we select the edge coverage point increased by the third expansion with the highest increment as the effective edge point. For large holes, we still select the edge coverage point increased by the third expansion as the effective edge point, and the corresponding edge points are shown in Figure 14.

**Figure 15:**
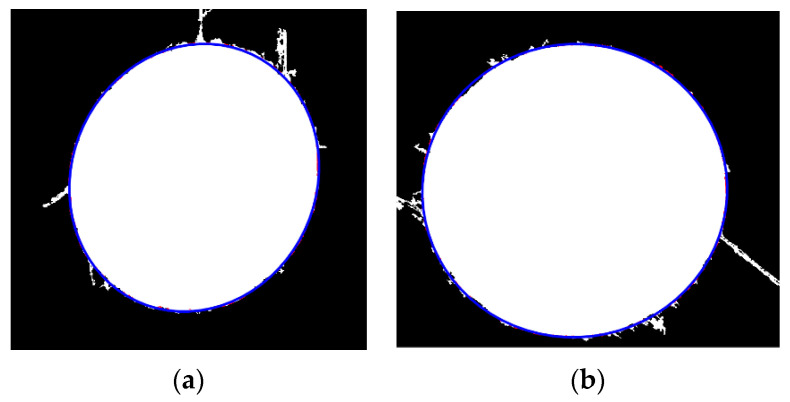
Ellipse fitting results. (**a**) Small hole; (**b**) large hole.

We performed the least squares ellipse fitting described in the second section on the elliptical hole after edge repair, and the fitting results are shown in Figure 15.

For the evaluation of the measurement results yielded by the method proposed in this article, we used the OGPMVP200 (Quality Vision International, Inc, Rochester, NY, USA) measuring instrument to fit the same measured holes, and compared them with the measurement results derived with the method proposed in this article. The measurement data obtained are shown in Table 2.

From Table 2, it can be seen that the inspection objects are all qualified products. The first row shows the results of the large and small holes measured by OGP. The first row, second column, and third column show the machining dimensions of the small circle and the fitting results of OGP, respectively. The first row, fourth column, and fifth column show the machining dimensions of the large circle and the fitting results of OGP, respectively. The second row shows the measurement results yielded by this method. The actual machining size of the small hole is 1.9 mm in diameter, with a tolerance of 0.05 mm. The machining inclination angle is 26°, and the actual machining size of the large hole is 2.3, with a tolerance of 0.05 mm. From the fitting results of the first line OCP, it can be seen that due to the presence of surface defects, the measurement results of the large and small holes obtained by the OGP measuring instrument are larger than the size of the workpiece. The diameters of the processed large and small circles are 1.9 mm and 2.3 mm, respectively, with a size error of about 0.1 mm and a maximum angle error of 0.8°. However, the algorithm proposed in this article produces results with a size error of less than 0.01 mm and an angle error of less than 0.15°, thus meeting the measurement requirements. From this, we can see the effectiveness and accuracy of the method proposed in this paper.

## 4. Conclusions

This article proposes a defect ellipse edge repair and fitting method based on vision for the rapid measurement of array micro oblique holes with defects such as scratches and burrs. Firstly, an edge repair algorithm is designed using the approximation principle of circles, and then the repair process is evaluated. Furthermore the repaired edges are obtained by firstly using morphological processing methods to obtain effective edge points of the ellipse to be fitted, which can effectively remove protruding outliers and concave outliers from the edge of the ellipse, and then use the least squares method is used to obtain effective ellipse parameters. This method is simple and effective in implementation, and can quickly obtain the parameters of defect ellipses. Through simulation and experiments, it can be determined that for oblique microwell images of 1920 × 1080 pixels, the dimensional variation measured by this method is less than 0.01 mm, and the angle error is less than 0.15°, which meets the accuracy requirements of an actual measurement. This method proposed in this article has a high measuring speed, which makes it suitable for engineering applications. In subsequent measurements, it can provide visual guidance for the precise measurement of fiber probes.

## Figures and Tables

**Table 1:** Defect ellipse fitting parameters.

Type of Ellipse	Center of a Circle (pix)	Long and Short Axes (pix)	Angle of Roll (°)
standard ellipse	(300, 400)	(600, 400)	0
protruding defect ellipse	(300.03, 400.50)	(597.94, 399.75)	−0.02
concave defect ellipse	(300.00, 400.14)	(599.45, 399.97)	−0.02
mixed defect ellipse	(300.28, 399.78)	(597.43, 398.89)	−0.09

**Table 2:** Ellipse machining parameters and fitting parameters.

Method	Parameter	Small Hole	Fitting Results	Large Hole	Fitting Results
OGP	short axis (mm)	1.9	1.9942	2.3	2.4147
	long axis (mm)	-	2.2119	-	2.5034
	angle of roll (°)	26	25.78	16	15.29
the method of this article	short axis (mm)	1.9	1.9027	2.3	2.3081
	long axis (mm)	-	2.1176	-	2.4025
	angle of roll (°)	26	26.04	16	16.11

## Data Availability

Not applicable.
